# Reef Fishes at All Trophic Levels Respond Positively to Effective Marine Protected Areas

**DOI:** 10.1371/journal.pone.0140270

**Published:** 2015-10-13

**Authors:** German A. Soler, Graham J. Edgar, Russell J. Thomson, Stuart Kininmonth, Stuart J. Campbell, Terence P. Dawson, Neville S. Barrett, Anthony T. F. Bernard, David E. Galván, Trevor J. Willis, Timothy J. Alexander, Rick D. Stuart-Smith

**Affiliations:** 1 Institute for Marine and Antarctic Studies, University of Tasmania, Hobart, TAS, Australia; 2 Stockholm Resilience Centre, Stockholm University, Stockholm, Sweden; 3 Wildlife Conservation Society, Indonesia Marine Program, Bogor, Indonesia; 4 School of the Environment, University of Dundee, Dundee DD1 4HN, Scotland, United Kingdom; 5 South African Environmental Observation Network Elwandle Node, Grahamstown, South Africa; 6 Zoology and Entomology Department, Rhodes University, Box 94, Grahamstown, South Africa; 7 Centro Nacional Patagónico–CONICET, Puerto Madryn, Chubut, Argentina; 8 School of Biological Sciences, Institute of Marine Sciences, University of Portsmouth, Portsmouth, United Kingdom; 9 Department of Fish Ecology and Evolution, Centre of Ecology, Evolution and Biogeochemistry, EAWAG Swiss Federal Institute of Aquatic Science and Technology, Kastanienbaum, Switzerland; 10 Division of Aquatic Ecology and Evolution, Institute of Ecology and Evolution, University of Bern, Bern, Switzerland; University of Windsor, CANADA

## Abstract

Marine Protected Areas (MPAs) offer a unique opportunity to test the assumption that fishing pressure affects some trophic groups more than others. Removal of larger predators through fishing is often suggested to have positive flow-on effects for some lower trophic groups, in which case protection from fishing should result in suppression of lower trophic groups as predator populations recover. We tested this by assessing differences in the trophic structure of reef fish communities associated with 79 MPAs and open-access sites worldwide, using a standardised quantitative dataset on reef fish community structure. The biomass of all major trophic groups (higher carnivores, benthic carnivores, planktivores and herbivores) was significantly greater (by 40% - 200%) in effective no-take MPAs relative to fished open-access areas. This effect was most pronounced for individuals in large size classes, but with no size class of any trophic group showing signs of depressed biomass in MPAs, as predicted from higher predator abundance. Thus, greater biomass in effective MPAs implies that exploitation on shallow rocky and coral reefs negatively affects biomass of all fish trophic groups and size classes. These direct effects of fishing on trophic structure appear stronger than any top down effects on lower trophic levels that would be imposed by intact predator populations. We propose that exploitation affects fish assemblages at all trophic levels, and that local ecosystem function is generally modified by fishing.

## Introduction

Marine protected area (MPA) networks represent an experimental set of ecological plots with reduced human extraction pressure. As such, MPAs provide an ideal focus for improved understanding of broad-scale effects of protection through comparison of differences in fished and protected areas [1]. Effective MPAs also provide a reference benchmark as undisturbed ecosystems for comparison with sites with high human impact [2]. Nevertheless, many MPAs do not appear to be effectively achieving conservation goals [3–6].

One widely predicted ecological consequence of fishing, which can be tested using MPAs, is that trophic reorganization occurs as a result of decreased top-down control from exploited populations of large fishes. According to this prediction, large predatory species that are disproportionately targeted by fishers should benefit more from MPAs than other groups, with effects of increased predation pressure cascading through the food web and variably affecting non-predatory species [7–10].

An example where an MPA was used to examine fish community restructuring comes from northeastern New Zealand, where protection was found to reduce the density of some small cryptic fish species within reserves with higher abundance of predators relative to nearby open-access areas [11]. Likewise, most prey species of the fishery-targeted coral trout (*Plectropomus* spp.) were less abundant in studied no-take zones in the Great Barrier Reef Marine Park that had high coral trout biomass [10]. Reduced abundance and biomass of fish prey species within MPAs were also noted in a global meta-analysis [7], indicative of possible indirect effects of competition or predation. On the other hand, a lack of prey abundance may limit predator growth [12] and abundance [13] demonstrating a bottom up effect on community structure. We hypothesized that a general control of top-down processes by fishing would result in a comparatively low biomass of smaller size classes of lower trophic groups within effective MPAs, in which carnivore biomass was known to be high relative to fished areas.

While predatory fishes are most heavily exploited, fishers can also target species in lower trophic groups such as herbivores, which may lead to reduced grazing of macroalgae with negative effects on live coral cover [14]. In the Caribbean, for example, the abundance of large parrotfishes increased in MPAs, which resulted in a doubling of grazing pressure on macroalgae, a major competitor of coral [15]. Such examples highlight the complex trophic changes that can follow MPA establishment, and the potential importance of fish size as well as trophic level when assessing trophic responses to fishing and protection. Other factors, independent from direct trophic cascades, can also play a role in shaping the fish community trophic structure [16]; spatial variability in recruitment, competition, isolation and oceanographic conditions can all contribute to the variation in biomass of the different trophic groups [3,17].

We use a global-scale dataset obtained with consistent survey methodology to test the generality of divergence in food webs in MPAs in relation to open-access sites as an indication of the impacts of fishing on trophic structure. We address the following specific questions:

1. Do different levels of MPA protection differentially affect the biomass of the four major fish trophic groups (higher carnivores, benthic carnivores, planktivores and herbivores)?
2. Do patterns in fish biomass partitioning among trophic groups across the global MPA network support the trends in trophic restructuring observed in some individual MPAs? Specifically, is there a general trend for reduced biomass of non-target species in effective MPAs where predator biomass is greater?

## Materials and Methods

**Fig 1:**
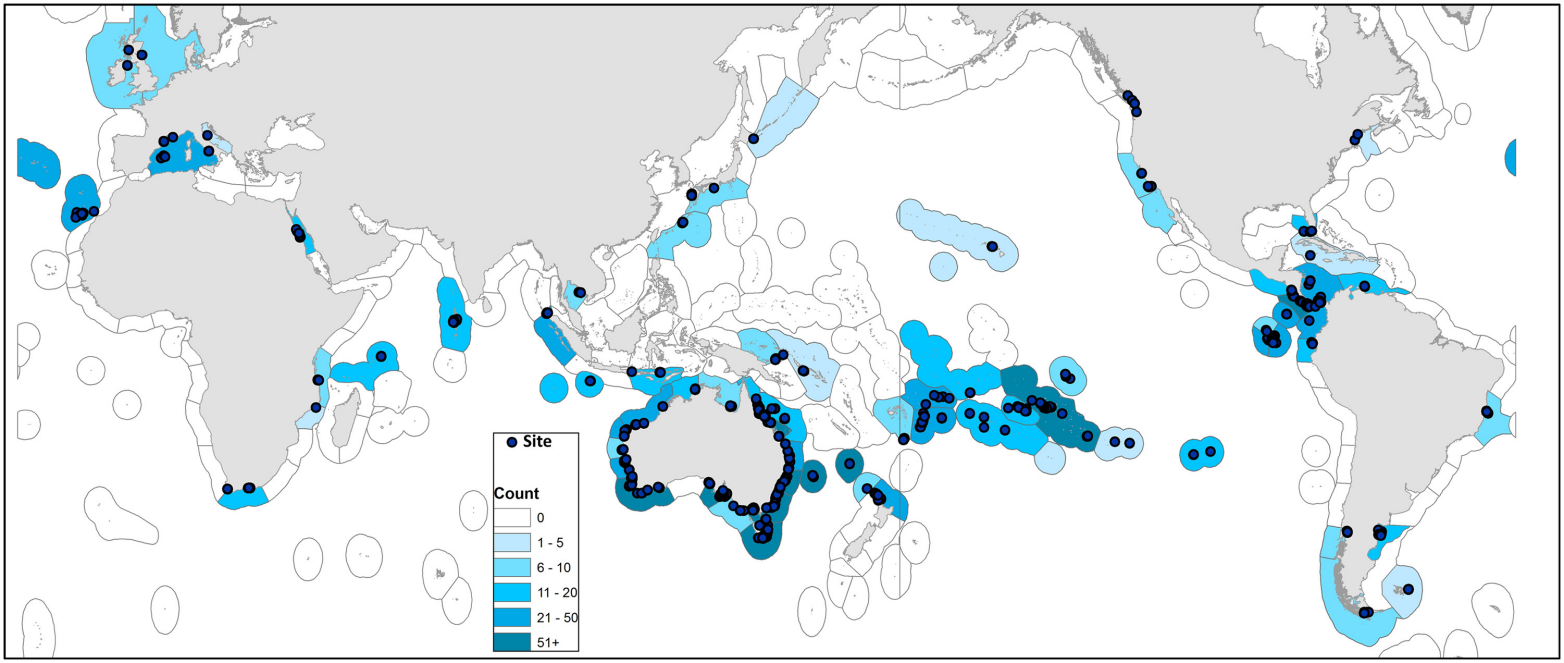
Global map showing sites investigated. The density of fill color applied to each marine ecoregion [19] relates to the number of sites surveyed within it.

Marine ecological survey data were collected worldwide through the Reef Life Survey program (RLS: www.reeflifesurvey.org) from September 2006 until November 2012 (see Edgar and Stuart-Smith [18] for details). The following authorities gave permission for field studies: Australia Department of Environment, Costa Rica Sistema Nacional de Areas de Conservacion, Galapagos National Parks Service, NSW Department of Primary Industries, New Zealand Department of Conservation, Panama Autoridad Nacional del Ambiente, Parks Victoria, Parques Nacionales Naturales de Colombia, Rottnest Island Authority, South Australia Department of Environment Water and Natural Resources, Tasmania Parks and Wildlife Service, United States Fish and Wildlife Service, United States National Park Service, Western Australia Department of Parks and Wildlife. Data covered 1,844 rocky and coral reef sites in 11 realms and 74 ecoregions [19] (Fig 1). The level of protection from fishing of each site was classed as no-take (no fishing allowed), restricted fishing (some form of fishery restrictions in place), or open-access. The ecological effectiveness of MPAs also depends on compliance with regulations and time since declaration [3]. Here we considered no-take zones to be effective if they exhibited a medium to high level of enforcement and had been established for at least five years prior to the fish survey (S1 Table). MPAs at which limited fishing was allowed, where enforcement of regulations was poor, and/or less than five years had elapsed since creation were considered less effective. Sites were assessed for effective protection using information on zoning in management plans, patrolling capacity, and infractions observed while in the field undertaking surveys, as described in Edgar et al. [3]. Open-access sites lay outside MPAs, or inside MPAs in zones with no restrictions on fishing other than national regulations (e.g. no explosives). A total of 79 MPAs were investigated, including some with multiple zones of differing effectiveness. A total of 405 sites within 50 MPAs were classified as effective no-take areas, 509 sites within 54 MPA were classified as low effectiveness, and 930 sites were open-access.

Fish species, abundance and size classes were surveyed using methods described in Edgar and Stuart-Smith [18]. In summary, divers laid a 50 m transect line and surveyed fishes within duplicate 5 m strips either side of the line (total area surveyed = 500 m^2^). All fish species present in each survey were identified, and their abundances and sizes estimated. Fish lengths were allocated into 2.5 cm bins to 15 cm, 5 cm bins between 15 and 40 cm, and to 12.5 cm bins for fish larger than 50 cm. Fish biomass was estimated using the abundance and sizes of fishes on transects and species-specific length-weight relationships provided in Fishbase. When length-weight relationships were unknown for a species, values were taken from a similarly-shaped relative. Fish surveys under the RLS program were conducted by both professional scientists and trained volunteer divers. Prior assessment of data quality showed no significant difference between these two groups [20]. Training of volunteer divers and data quality control processes are outlined in Edgar and Stuart-Smith [18].

For data analyses, fishes were divided into four major trophic groups: higher carnivores, benthic carnivores, planktivores and herbivores, based on dietary information obtained from Fishbase (www.fishbase.org). If insufficient information was available for a particular species, the closest relative was used as a proxy. Higher carnivores were those fishes with diets primarily composed of other fishes, decapods and cephalopods. Benthic carnivores fed predominantly on invertebrate fauna, most commonly peracarid crustaceans, molluscs, polychaetes, sponges or corals. Herbivorous species included all fishes for which algal food sources formed a major part of the diet. This group included detritivorous and omnivorous species, as well as scraping and excavating parrotfishes. It thereby covered a diverse range of more specialized trophic groups and functional roles [21].

Regardless of trophic level, larger fishes are less likely to be negatively affected through predation by large carnivores. Thus, trophic groups were also sub-categorized into three size classes: small (<7.5 cm), medium (7.5–30 cm) and large (>30 cm), depending on the observed size of fishes during the surveys.

### Statistical analysis

Linear Mixed Models (LMMs) were applied using all sites within an ecoregion to compare the effect of protection, adjusting for five important environmental and anthropogenic covariates. These covariates represented factors found to influence the spatial patterns of biomass in prior analyses [3,22]. Environmental data, including annual mean sea surface temperature (SST), SST range and photosynthetically-active radiation (PAR-mean), were extracted from Bio-Oracle [23] (S2 Table). PAR-mean was calculated by averaging daily PAR for each month and then across the year [23]. A human population index (Pop index) was calculated by fitting a smoothly tapered surface to each settlement point created with the glp00g gridded world population density dataset (http://sedac.ciesin.columbia.edu/data/collection/gpw-v3/sets/browse). The quadratic Kernel function was applied, as described in Silverman [24]. Populations were screened to only include populations with density greater than 1000 people per 0.04° cell. The values did not directly represent the population values since they were both modelled (quadratic) and smoothed. However, these values provide a comparative index of population density/pressure.

**Fig 2:**
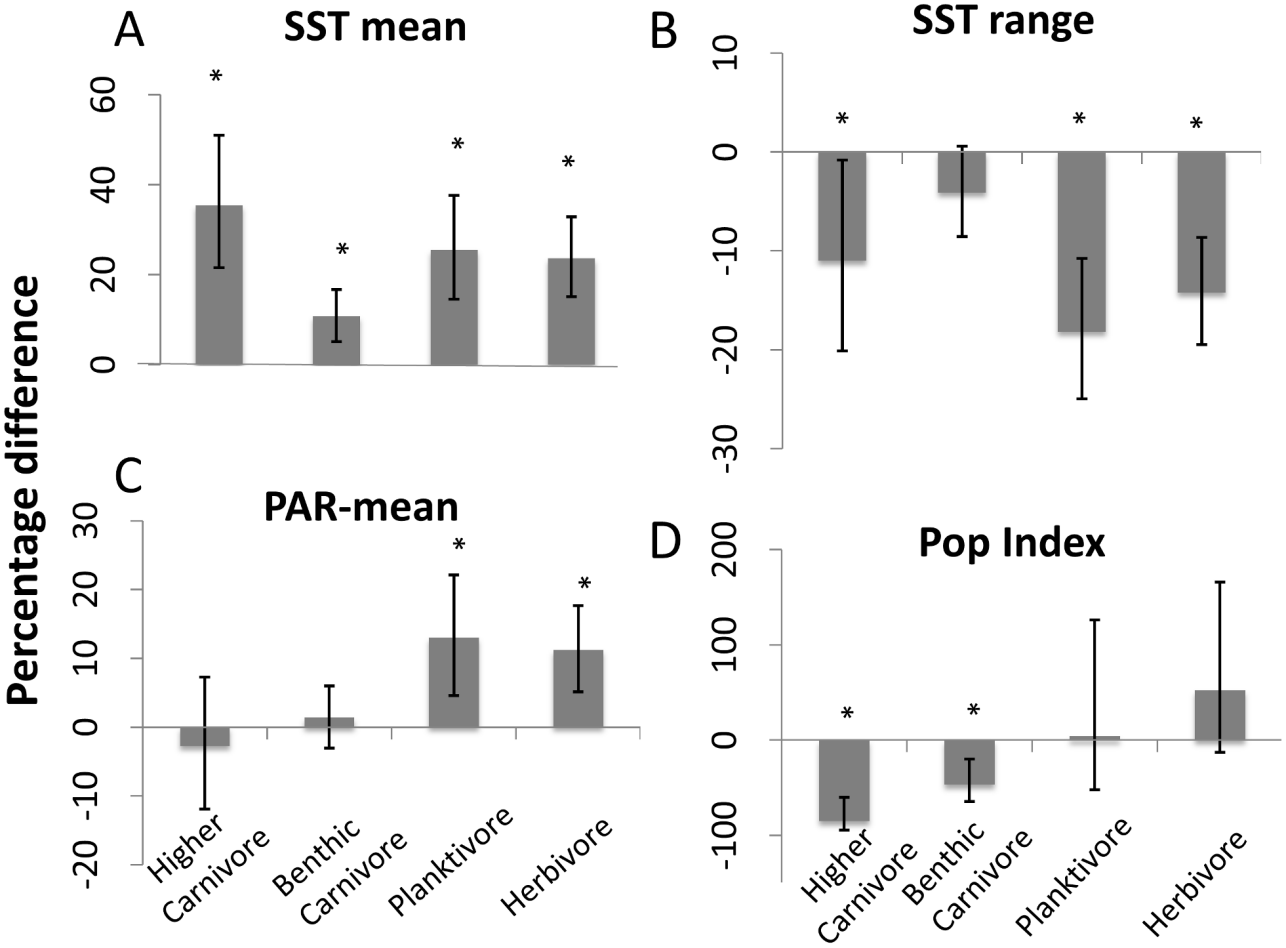
Percentage difference in biomass (± 95% confidence intervals) for covariates investigated. Percentage difference in biomass per 1°C change in mean sea surface temperature (A), 1°C change in the annual range in sea surface temperature (B), 1 Einstein/m^2^/day change in annual mean photosynthetically-active radiation (C), and a single unit increase in the index of local population density (D) for each of the four trophic groups. Ratios were obtained from the *β* coefficients and transformed into % increment in biomass, by 100*(exp(*β*)-1). Asterisks denote statistically significant difference (p<0.05).

Of ten environmental and anthropogenic covariates examined (S2 Table), SST mean, SST range, PAR-mean and the population index had the greatest influences on the biomass of the four trophic groups (Fig 2). Consequently, analyses of MPA effects first accounted for these four factors, plus the random effects of site nested hierarchically within ecoregion [19], which in turn was nested within realm.

The effect of MPA protection on the different trophic groups was assessed using LMMs, with MPA protection introduced after the influences of other variables (SST mean, SST range, PAR, Pop index) were considered in models. This allows us to test the influence of protection while considering other factors, which affected the observed biomass of the different trophic groups. This same model was also applied to test for differences in biomass of the size classes of different trophic groups:

$$y_{rei\;}=\;\mu \;+\;\beta {\text{1SSTmean}}_{i}+\;\beta {\text{2SSTrange}}_{i}+\;\beta {\text{3PARmean}}_{i}+\;\beta {\text{4POPindex}}_{i}+\;\beta {\text{5Protection}}_{i}+\;\delta_{r}\;+\;\gamma_{re}\;+\;\varepsilon_{rei\;}$$ (1)

where *y*_*rei*_ = log (total biomass of fishes + 100, in g) at the *i*th site, given the effects of SST mean, SST range, PAR-mean and human population, conducted in the Ecoregion *e* and Realm *r*; *μ* = overall mean; *β*_*1*,*2*,*3*,*4*,*5*_ = effect of SST mean (*1*), SST range (*2*), PAR mean (*3*) and Pop index (*4*) and Protection (*5*) on the mean; *δ*_*r*_ = effect of the *r*th realm; *γ*_*re*_ = effect of the *e*th ecoregion within the *r*th realm (both realm and ecoregion are random effects); *ε*_*rei*_ = residual error. Due to the absence of some trophic groups in some sites surveyed, we added a constant (= 100) to all raw fish biomass [ln(y+100)]. Given that biomass was scaled in grams, the addition of 100 g to the transect was chosen as a reasonable ecological value for the step between no biomass and minimum observed biomass [25]. A 4^th^ root transformation of biomass was also tested, and provided similar outcomes as the log transformation. Results from the log transformation are presented here so that the effect size can be presented as % difference in biomass.

Effects of the two effectiveness categories of MPAs relative to open-access areas were estimated within LMMs by estimating the log ratios of biomass (log(biomassMPA/biomassOPEN)). These were obtained from the coefficient for Protection, *β*_*5*_ and were transformed into % increment in biomass, by 100*(exp(*β*_*5*_)-1). P-values generated were based on the Wald statistic. Likelihood ratio tests (LRT) were also applied; nevertheless, due to the very large sample size, conclusions were the same as with the LMMs, so LRT results are not additionally presented here.

Numerous ecoregions did not have sites within both MPAs and open-access areas. Consequently, additional LMMs were constructed with a subset that included the reduced set of 35 ecoregions that contained representatives of two zone types (protected areas vs open-access zones). Similar results were found with this subset of the data compared with the results obtained using the whole data set; therefore, results presented were based on the complete data set.

All statistical analyses were performed in R-Studio using the package nlme [26].

## Results

The population index was used as a proxy for human pressure, and had a significant negative effect on biomass of higher and benthic carnivores (Fig 2). The three environmental covariates, SST mean, SST range and PAR-mean, also had significant effects on fish biomass. However, only SST mean was significant for all four trophic groups (Fig 2). PAR-mean had a significant positive effect for biomass of planktivores and herbivores. SST range had a significant negative effect on biomass of higher carnivores, planktivores and herbivores (Fig 2).

**Fig 3:**
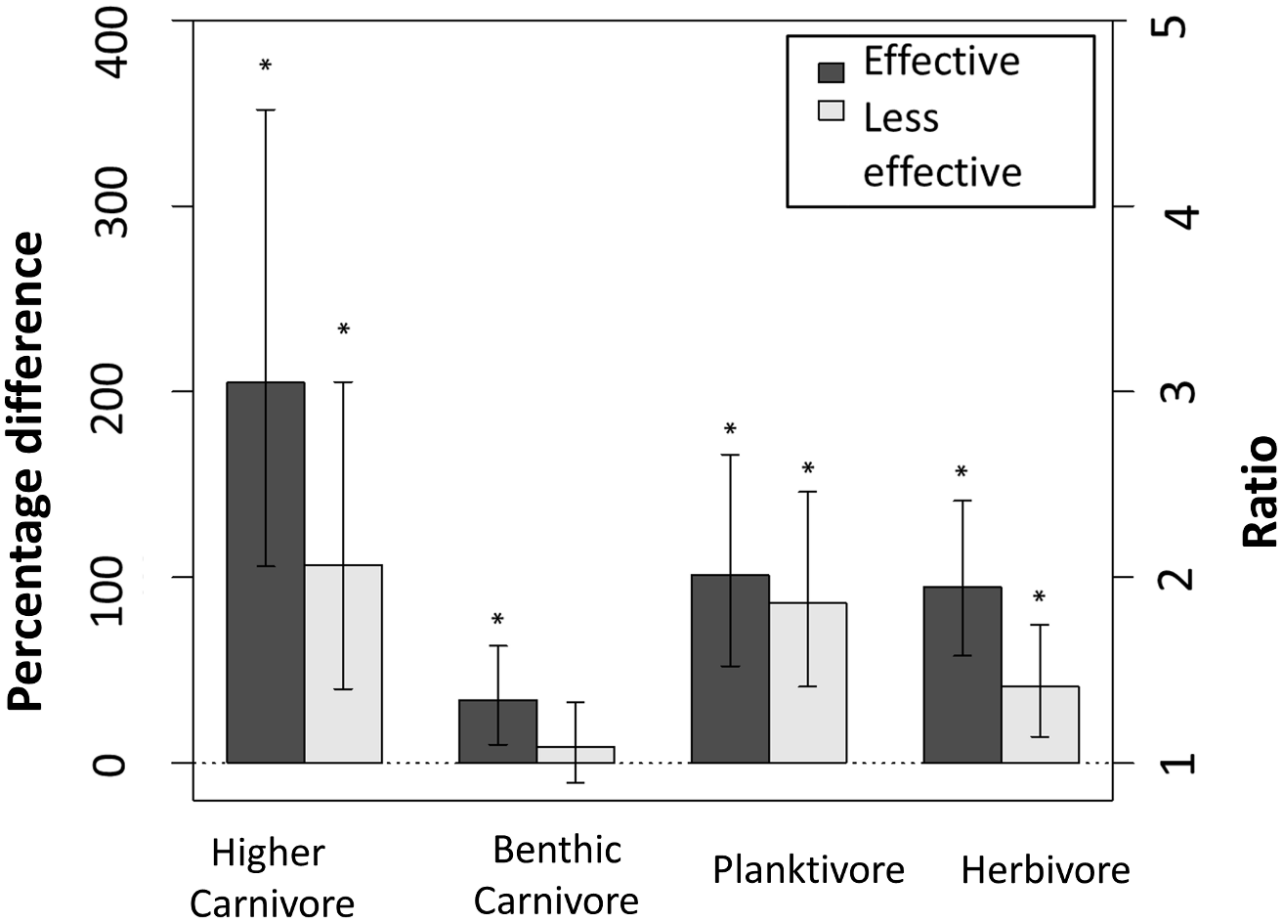
Percentage difference in biomass of different trophic groups in protected areas relative to open-access zones. Log ratios of biomass (log(biomassMPA/biomassOPEN)) between effective and less effective MPAs relative to open-access zones, for each trophic group (± 95% confidence intervals). Ratios were obtained from the coefficient for Protection, *β*_*5*_, in Eq 1 and transformed into percentage increments in biomass, by 100*(exp(*β*_*5*_)-1). Asterisks denote a statistically significant difference (p<0.05). The model also adjusted for SST mean, SST range, PAR-mean and human population.

Protection from fishing clearly affected reef fish community structure. All trophic groups possessed significantly higher biomass in effective MPAs compared to open-access areas (Fig 3). The biomass of higher carnivores, herbivores and planktivores were also significantly higher in less effective MPAs compared to open-access areas (Fig 3), and differences between effective MPAs and less effective MPAs were only non-significant for planktivores.

**Fig 4:**
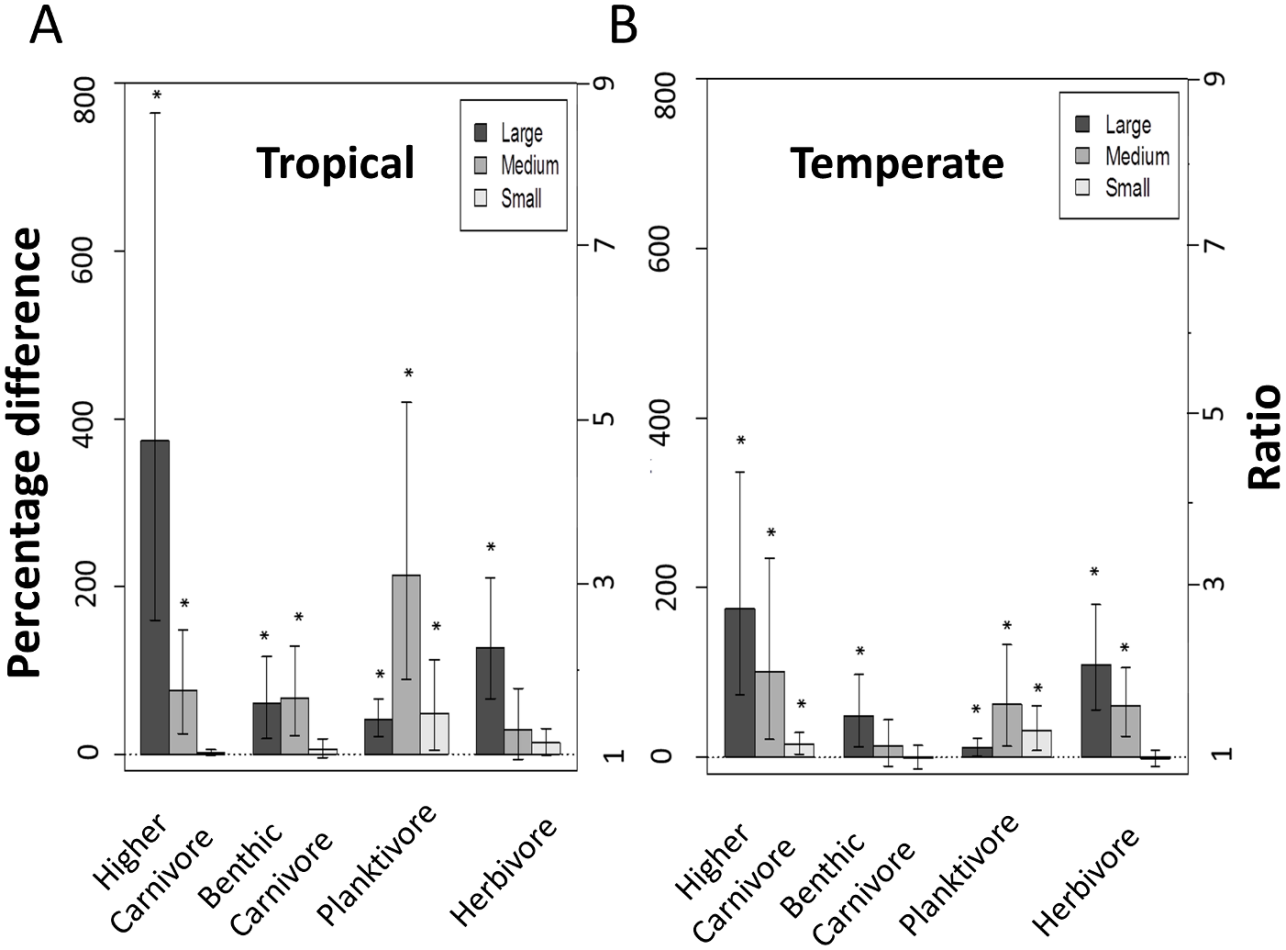
Percentage difference in biomass of the different trophic groups size categories in temperate and tropical areas. Log ratios of fish biomass (log(biomassMPA/biomassOPEN)) between trophic and body size groups in effective MPAs in temperate (A) and tropical (B) regions, relative to open access zones (± 95% confidence intervals). Small fishes <7.5 cm; medium fishes from 7.5 to 30 cm; large fishes >30 cm. Ratios were obtained from the coefficient for Protection, *β*_*5*_, and transformed into percentage increments in biomass, by 100*(exp(*β*_*5*_)-1). Asterisks denote a statistically significant difference (p<0.05). The model also adjusted for SST mean, SST range, PAR-mean and human population.

Differences in fish biomass between effective MPAs and open-access areas were remarkably consistent for different fish size classes and trophic groups between tropical and temperate realms (Fig 4). Biomass of large fishes (maximum length >30 cm) was significantly higher than in open-access areas for all four trophic groups, in both tropical and temperate zones. Biomass of medium-sized fishes (7.5–30 cm) in effective MPAs was also higher for most of the trophic groups in the tropics and temperate regions, with the exception of medium-sized benthic carnivores in temperate zones and medium-sized herbivores in the tropics. For the small size classes (<7.5 cm), planktivores were recorded in significant higher biomass in effective MPAs in the tropics. Small higher carnivores and planktivores exhibited significant higher biomass in effective temperate MPAs (Fig 4). Although some groups exhibited similar biomass across MPAs and open-access areas, no size classes of any trophic group had significantly lower biomass in effective MPAs relative to open access areas.

## Discussion

Our results show clear differences in fish community structure due to protection from fishing in effective MPAs (i.e. no-take, with medium to high enforcement and over 5 years old). Higher biomass of large predatory species was particularly marked, while the biomass of all other trophic groups was either greater or equal in effective MPAs compared with open-access sites. Thus, none of the trophic or size groups had negative biomass ratios in MPAs compared with open-access sites that would indicate patterns for top-down control of the fish community in the presence of greater biomass of predatory fishes. This result was consistent even when considering only the MPAs identified as most effective and having the five attributes of protection outlined in Edgar et al. [3] and known to have elevated biomass of the major groups of predatory and exploited species. This does not imply that cascading trophic interactions have not occurred in effective MPAs, as our study did not look at individual species, but rather at the scale of whole trophic size groups. However it does present two clear, novel outcomes with respect to general MPA effects: (1) that protection from fishing appears to favor all trophic groups, some much more than might be expected (e.g. medium-sized planktivores); and (2) that regardless of whether cascading trophic interactions occur within MPAs, the disparity in the biomass ratios between MPAs and open access sites for the different size-classes of trophic groups implies a trophic re-organisation that is likely to have substantial consequences for ecological functions.

### Higher biomass in all trophic groups

Our findings are also in accordance with previous studies [7,27] that show higher fish biomass in effective MPAs [28]. As predicted, the greatest difference involved large higher carnivores. However, none of the trophic groups showed lower biomass in MPAs in relation to open-access sites, implying that impacts of exploitation across marine food webs may often be underestimated.

Preferential targeting of large herbivorous species has been well documented in many tropical regions [14,15], and is opposite to the effect of the fishing down the food web [28]. We suggest that while large-scale commercial fisheries, which operate in deep and pelagic offshore waters, may often first remove higher trophic level species, exploitation of reef species from shallow, coastal waters, including artisanal fisheries and recreational anglers, is less trophically-selective. If correct, then caution is needed when applying the widely used Marine Trophic Index [29] as an indicator for fishing impacts in shallow reef habitats. A highly consistent response among larger size classes of all trophic groups supports hypotheses that fishing impacts are more size-based than focused on particular trophic groups.

Despite the apparently greater importance of size than trophic group, differences of biomass between effective MPAs and open-access sites were unequal across trophic groups. The biomass difference was greatest for large higher and benthic carnivores in general, with a more pronounced difference in effective MPAs. The simplest explanation is that fishing has had the greatest impact on these groups outside effective MPAs. This is supported by a significantly lower biomass of carnivorous fishes in locations with highest human population density (Fig 2). Nevertheless, at least two other mechanisms associated with protection could also potentially lead to this result: (1) biomass recovery may be faster for large carnivorous fishes, and (2) predation pressure by large higher carnivores and benthic carnivores may limit potential increases in biomass of other groups, rather than reducing their biomass.

The first of these two alternative explanations is plausible, given that individual growth is generally rapid in piscivorous fishes, and that many of the effective MPAs studied were still young relative to the time required for growth of individual fishes. Only 40% of effective MPAs investigated were more than 10 years old, and thus much of the recorded differences in biomass is likely to be associated with direct recovery of fished species [30,31].

A short time frame also supports the second alternative explanation, in that predator biomass is still likely increasing and may not yet have reached the point where prey biomass suppression is evident. Furthermore, top down control may never manifest if the impact of fishing on smaller sizes and/or lower trophic groups is greater than predation pressure from large predatory fishes alone. Thus, in some cases the top-down pressure from humans may be of much greater magnitude than that exerted by predatory fishes in completely unexploited communities.

Our results likely represent a combination of the two potential mechanisms described above, with additional complexity added by recruitment, competition, oceanographic conditions, isolation, predation from higher vertebrates and invertebrates, and habitat structure. With respect to habitat structure, we conducted additional analyses using a subset of sites (482) for which we had data on the structure of the reef, scored using an index of vertical relief (S1 Appendix). Higher relief index values were associated with elevated biomass of higher carnivores, benthic carnivores and planktivores (S1 Fig), confirming the importance of structural complexity in supporting greater fish biomass in general [32]. After accounting for complexity in this subset of sites, the effect of protection remained consistent with those from analysis of the full dataset (S2 Fig). Thus, both protection and relief have significant effects on the biomass of fish independently, but our conclusions relating to MPA effects are unlikely confounded by habitat complexity.

Another potential source of bias is that faster moving fish are typically oversampled in underwater visual censuses [33]. This should not affect conclusions if the bias is systematic between fished and unfished locations, but if behavioural patterns change in MPAs, with attraction to divers, then the magnitude of difference between fished and unfished locations for large carnivores will be overstated.

When differences in fish biomass between MPAs and open-access sites across the various size and trophic groups are considered together, our data suggest that protected reef fish communities probably function quite differently to those in fished locations. As shown in Fig 4, larger carnivorous fishes are present in proportionally greater biomass in effective MPAs compared to open-access sites, and this is likely to have important ecological implications. For example, a substantial shift to larger herbivores may increase resilience in coral reef locations [34], while recovery of large predators in a temperate MPA has been hypothesized to contribute to ecosystem resistance to tropicalisation [35].

### Other drivers of reef fish trophic structure

SST mean was positively related to the biomass of the four trophic groups, which aligns with the latitudinal gradient in total fish biomass [36,37]. Furthermore, SST range was negatively related to the biomass of three of the trophic groups. High variation in SST throughout the year is typical of high latitudes and sheltered embayments [38,39]. Interestingly, benthic carnivores were least affected by extreme seasonality, possibly reflecting greater stability in food sources in such areas, or possibly more varied generalist behavioral and feeding strategies within this very broad group of fishes. Biomass of planktivores and herbivores showed a significant positive relationship with PAR-mean, as would be expected based on increased productivity of benthic algal and phytoplankton-driven food sources [40].

A trend for decreasing fish biomass with increasing human population density is an increasingly common finding of broad-scale studies [3,17,30,41,42]. Our results expand on prior results by suggesting a greater negative impact on carnivorous species than herbivores or planktivores when examined at the global level. This result, and the substantial variability in the effect of human population density on herbivorous and planktivorous species, likely reflects stronger regional inconsistencies in exploitation of these two groups, as well as patchy impacts associated with habitat degradation near population centers. Other factors that potentially contributed to observed results, but were not considered in this study, include the possible increase in other predators such as seals [43] and lobsters [30] in effective MPAs.

In conclusion, effective MPAs provide protection for multiple components of food webs, not just larger carnivorous fishes. General trends of top-down control by larger predator fish on smaller fish were less pronounced in our global analysis than prior reports for particular species at some individual MPAs. Elevated biomass of particular trophic and size groups will inevitably result in variability of local ecological processes. Human impacts on reef fish community structure were inferred to be stronger than top-down control by the larger predatory species when considered at the global scale; however, more time is needed for fish communities within the global MPA network to re-organize to the point where indirect trophic effects of fishing are strongly defined.

## Supporting Information

S1 Appendix Assessment of potential confounding of MPA effects by variation in habitat complexity. (DOCX)

S1 Fig Percentage difference in biomass (± 95% confidence intervals) for relief index. Percentage difference in biomass for 1 unit increase in the relief index (range 1–4) for each of the four trophic groups. The ratios were obtained from the coefficient for Relief, *β*_*5*_, from the LMM equation (S1 Appendix) and transformed into percentage increments in biomass, by 100*(exp(*β*_*5*_)-1). Asterisks denote a statistically significant difference (p<0.05).
(TIF)

S2 Fig Percentage difference in biomass (± 95% confidence intervals) of the different trophic groups in protected areas relative to open-access zones when accounting for, and not accounting for, the relief index. Log ratios of biomass (log(biomassMPA/biomassOPEN)) with relief index and without relief index included in the LMMs. The difference in biomass in effective MPAs were relative to open-access zones, for each trophic group (± 95% confidence intervals). Ratios were obtained from the coefficient for Protection, *β*_*6*_, from the LMM equation (S1 Appendix) and transformed into percentage increments in biomass, by 100*(exp(*β*_*6*_)-1). Asterisks denote a statistically significant difference (p<0.05). The LMM model also adjusted for SST mean, SST range, PAR-mean and human population.
(TIF)

S1 Table Marine Protected Areas (MPAs) level of effectiveness and number of sites. Some MPAs had sites that differed in level of effectiveness.
(DOCX)

S2 Table Covariates used as predictors in linear mixed models. PAR-mean, Nitrate, Phosphate, Silicate, Chlomean, SST range and SST mean were obtained from Bio-ORACLE [23]. Pop index was calculated using the quadratic kernel function described by [24].
(DOCX)
