# Supplementary material for: Reef Fishes at All Trophic Levels Respond Positively to Effective Marine Protected Areas
Source: PLoS One. 2015 Oct 13;10(10):e0140270. doi: 10.1371/journal.pone.0140270 (PMC4603671; doi:10.1371/journal.pone.0140270)
Supplement: S1 Appendix — (DOCX) [file pone.0140270.s001.docx]

**S1 Appendix**

*Assessment of potential confounding of MPA effects by variation in habitat complexity*

A relief index, a proxy for habitat complexity, was included as an extra covariate for the subset of 482 sites for which this information was available. The relief index was based on the size of boulders, crevices and general variation in reef height within the area surveyed. The relief index ranged from one to four, and was subjectively scored for the 482 sites by two of the authors during fish surveys. A score of one was given when the substrate was relatively flat (<0.5 m elevation), with few hiding places suitable for fishes, while a score of four indicated high complexity in the structure (i.e. large boulders and deep crevices) varying by >2 m in vertical height. The sites with relief information were located both in MPAs (239 sites) and open-access sites (243).

In our main analyses, the effect of MPA protection on the different trophic groups was assessed using Linear Mixed Models (LMMs), with MPA protection introduced after the influences of other variables (SST mean, SST range, PAR, Population index) were considered in the models. To test for the effect of complexity on our MPA effects, and therefore whether our major conclusions could be confounded by unaccounted for effects of complexity, we included relief as a fifth factor in LMMs for the subset of 482 sites. The model used is explained below:

*y_rei_* = *μ* + *β_1_* SSTmean*_i_*+ *β_2_* SSTrange*_i_*+ *β_3_* PARmean*_i_*+ *β_4_* POPindex*_i_*+ *β_5_*Relief +*β_6_*Protection + *δ_r_* + *γ_re_* + *ε_rei_*

where *y_rei_* = log (biomass of fish + 100) at the *i*th site, given the effects of SST mean, SST range, PAR-mean, human population and Relief, conducted in the Ecoregion *e* and Realm *r*; *μ* = overall mean; *β_1,2,3,4,5_* _,6_= effect of SST mean (*1*), SST range (*2*), PAR mean (*3*) and Pop index (*4*), Relief (*5*) and Protection (*6*) ; *δ_r_* = effect of the *r*th realm; *γ_re_* = effect of the *e*th ecoregion within the *r*th realm (both realm and ecoregion are random effects); *ε_rei_* = residual error.

The relief index had a positive effect on biomass of higher carnivores, benthic carnivores and planktivores at the subset of 482 sites (S1 Fig.). The difference in biomass due to the increase of one unit of relief (range from 1-4) was obtained from the coefficient for Relief, *β_5_*, from the LMMs and transformed into % increment in biomass, by 100*(exp(*β_5_*)-1.

Thus, both relief and protection were independently related to higher biomass of all trophic groups. The magnitudes of the effect of protection were similar after adjusting for relief, with the effect of protection remaining positive for all trophic groups (S2 Fig.).
