# Supplementary figures and images for: Reef Fishes at All Trophic Levels Respond Positively to Effective Marine Protected Areas
Source: PLoS One. 2015 Oct 13;10(10):e0140270. doi: 10.1371/journal.pone.0140270 (PMC4603671; doi:10.1371/journal.pone.0140270)

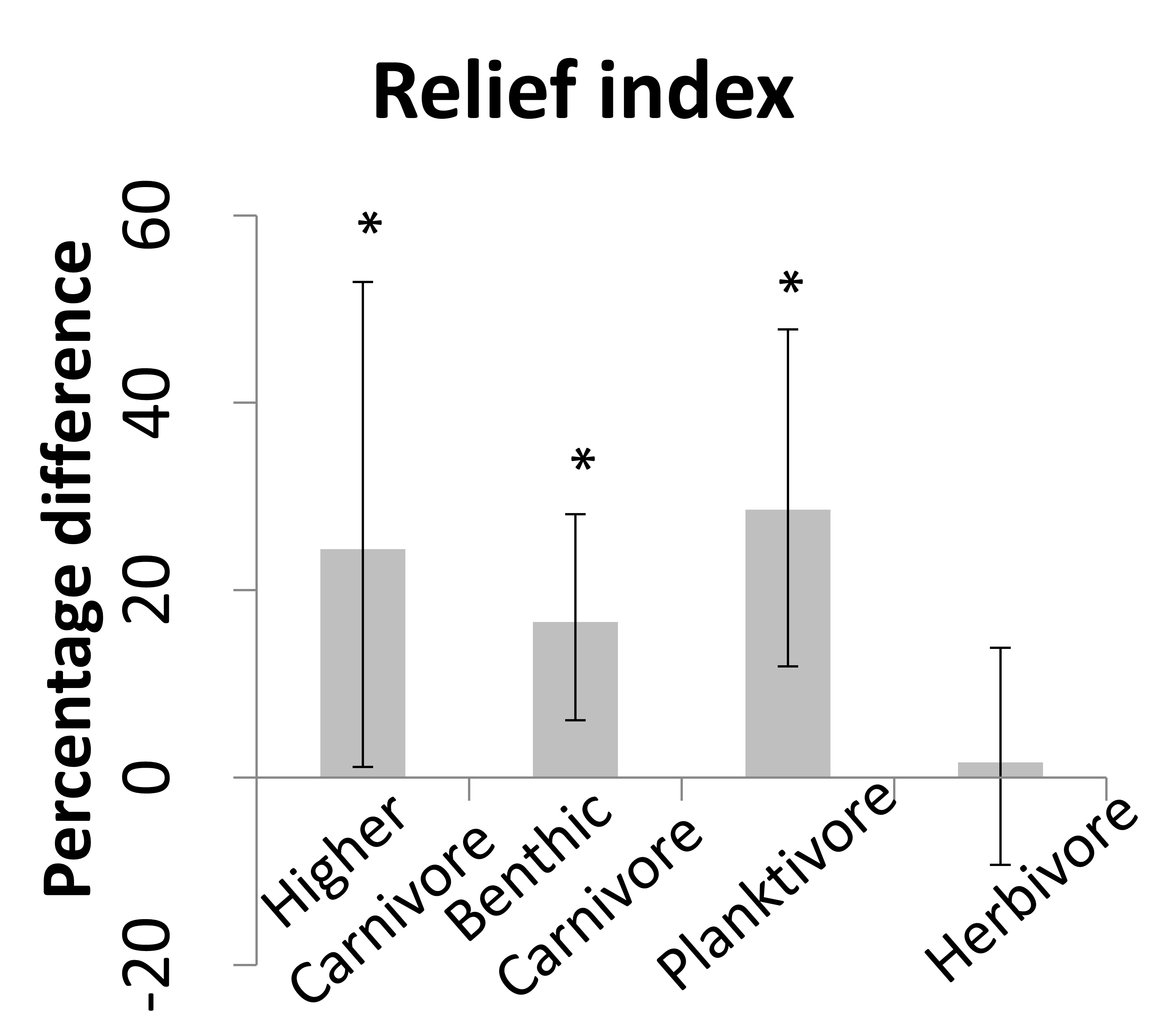

Supplement: S1 Fig — Percentage difference in biomass for 1 unit increase in the relief index (range 1–4) for each of the four trophic groups. The ratios were obtained from the coefficient for Relief, β 5, from the LMM equation (S1 Appendix) and transformed into percentage increments in biomass, by 100*(exp(β 5)-1). Asterisks denote a statistically significant difference (p<0.05). (TIF) [file pone.0140270.s002.TIF]

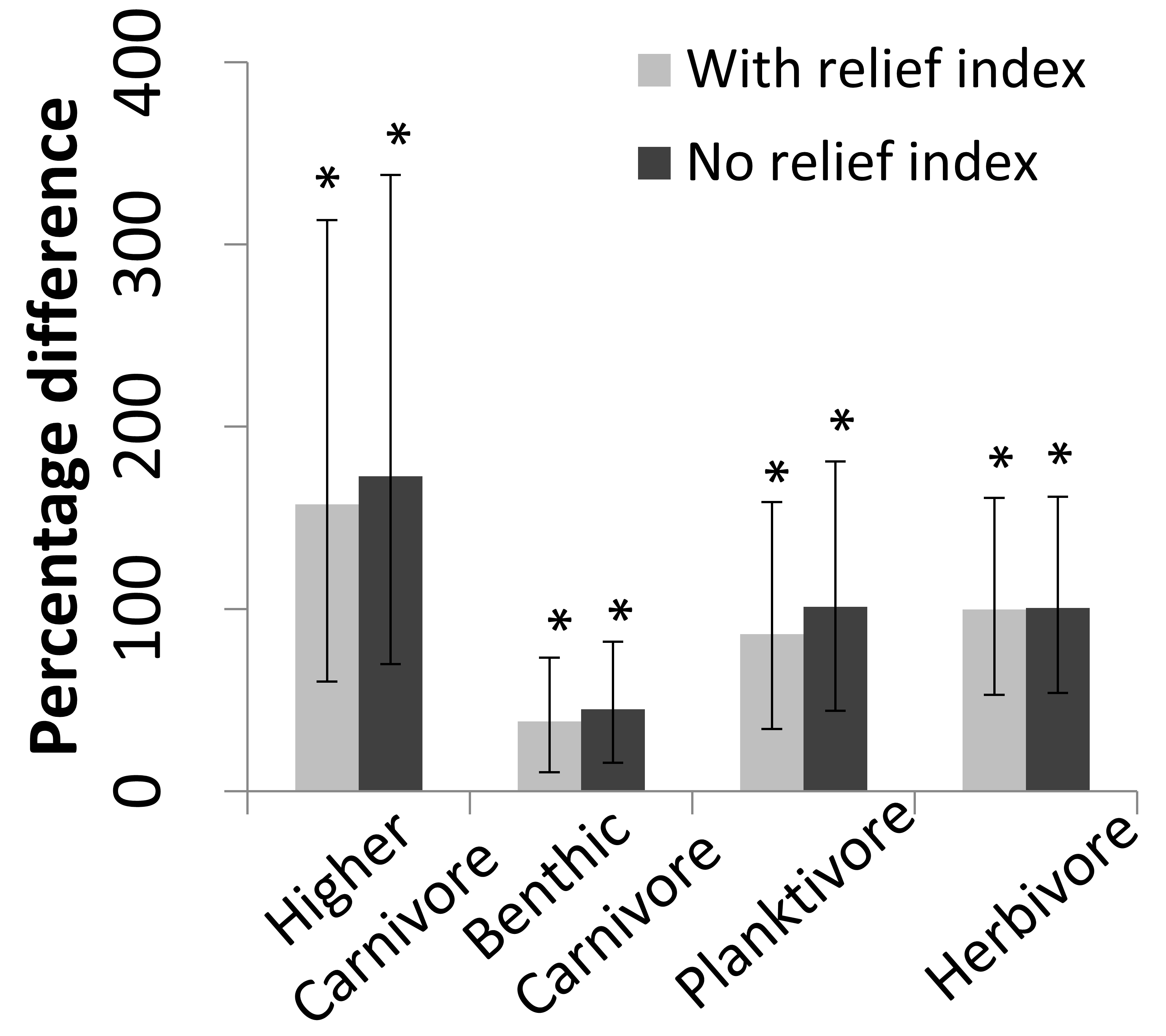

Supplement: S2 Fig — Log ratios of biomass (log(biomassMPA/biomassOPEN)) with relief index and without relief index included in the LMMs. The difference in biomass in effective MPAs were relative to open-access zones, for each trophic group (± 95% confidence intervals). Ratios were obtained from the coefficient for Protection, β 6, from the LMM equation (S1 Appendix) and transformed into percentage increments in biomass, by 100*(exp(β 6)-1). Asterisks denote a statistically significant difference (p<0.05). The LMM model also adjusted for SST mean, SST range, PAR-mean and human population. (TIF) [file pone.0140270.s003.TIF]
