# Supplementary material for: Reef Fishes at All Trophic Levels Respond Positively to Effective Marine Protected Areas
Source: PLoS One. 2015 Oct 13;10(10):e0140270. doi: 10.1371/journal.pone.0140270 (PMC4603671; doi:10.1371/journal.pone.0140270)
Supplement: S1 Table — Some MPAs had sites that differed in level of effectiveness. (DOCX) [file pone.0140270.s004.docx]

**S1 Table.** Marine Protected Areas(MPAs) level of effectiveness and number of sites. Some MAPs

| **Name MPA** | **Effective** | **Less effective** | **No. of sites** |
| --- | --- | --- | --- |
| APA Ponta da Baleia-Abrolhos | 6 | 4 | 10 |
| Baie Ternay | 2 |  | 2 |
| Batemans Marine Park | 6 | 50 | 56 |
| Beacon Island Reef Observation Area | 1 | 2 | 3 |
| Beware Reef Marine Sanctuary | 8 | 2 | 10 |
| Bonaire |  | 14 | 14 |
| Bronte-Coogee Aquatic Reserve |  | 3 | 3 |
| Bushrangers Bay Aquatic Reserve | 1 |  | 1 |
| Cabbage Tree Bay Aquatic Reserve | 3 | 1 | 4 |
| Caletas |  | 3 | 3 |
| Camaronal |  | 3 | 3 |
| Cape Banks Aquatic Reserve |  | 2 | 2 |
| Cape Byron Marine Park |  | 3 | 3 |
| Cape Howe Marine National Park | 4 | 5 | 9 |
| Cape Rodney _ Okakari Point Marine Reserve | 4 |  | 4 |
| Channel Islands National Marine Sanctuary | 6 | 7 | 13 |
| Cocos National Park | 23 |  | 23 |
| Coiba National Park | 26 | 22 | 48 |
| Coral Patches Reef Observation Area | 2 |  | 2 |
| Coringa-Herald Nature Reserve |  | 10 | 10 |
| Fiordo Comau Marine and Coastal Protected Area |  | 4 | 4 |
| Florida Keys National Marine Sanctuary | 12 | 5 | 17 |
| Fly Point-Halifax Park | 4 | 3 | 7 |
| Galapagos Marine Reserve | 42 | 27 | 69 |
| Golfo de Chiriqui Marine National Park |  | 4 | 4 |
| Governor Island Marine Nature Reserve | 1 | 4 | 5 |
| Great Barrier Reef MP | 43 | 28 | 71 |
| Hanauma Bay Marine Life Conservation District | 2 |  | 2 |
| Illa del Toro |  | 1 | 1 |
| Isla de Taboga e Isla de Uraba Wildlife Refuge |  | 3 | 3 |
| Jawbone Marine Sanctuary |  | 2 | 2 |
| Jervis Bay | 15 | 39 | 54 |
| Jurien Bay | 2 | 4 | 6 |
| Kawasan Wisata |  | 17 | 17 |
| La Reserve Naturelle Marine de Cerbere Banyuls | 4 | 6 | 10 |
| La Restinga-Mar de las Calmas MPA | 3 |  | 3 |
| Las Perlas Marine Special Management Zone |  | 25 | 25 |
| Leo Island Reef Observation Area | 1 |  | 1 |
| Levante de Mallorca Cala Ratjada |  | 1 | 1 |
| Lord Howe Island Marine Park | 26 | 25 | 51 |
| Machalilla |  | 13 | 13 |
| Malpelo Flora and Fauna Sanctuary | 11 |  | 11 |
| Maria Island Marine Reserve | 4 |  | 4 |
| Marmion Marine Park | 1 | 3 | 4 |
| Mnazi Bay-Ruvuma Estuary Marine Park |  | 1 | 1 |
| Motu Motiro Hiva |  | 8 | 8 |
| Mushi Mas Mingili Thila | 1 |  | 1 |
| Ninepin Point Nature Marine Reserve |  | 2 | 2 |
| Ningaloo Marine Park | 13 | 22 | 35 |
| North Sydney Harbour Aquatic Reserve |  | 14 | 14 |
| Pangaimotu Reef MPA | 1 |  | 1 |
| Panglima Laut |  | 4 | 4 |
| Point Cooke Marine Sanctuary |  | 1 | 1 |
| Point Lobos State Marine Reserve |  | 1 | 1 |
| Poor Knights Island Marine Reserve | 16 |  | 16 |
| Port Davey Marine Reserve | 3 |  | 3 |
| Port Noarlunga Reef | 1 |  | 1 |
| Port Phillip Heads Marine National Park | 22 | 10 | 32 |
| Port Stephens Great Lake Marine Park | 3 | 41 | 44 |
| Regno di Nettuno |  | 8 | 8 |
| Rickett's Point Marine Sanctuary | 2 |  | 2 |
| Rottnest Island | 3 | 20 | 23 |
| Seaflower Area Marina Protegida |  | 15 | 15 |
| Sesoko Scientific Research Area | 1 |  | 1 |
| Shiprock Aquatic Reserve | 1 |  | 1 |
| Shiraiwazaki Marine Park | 1 |  | 1 |
| Solitary Islands Marine Park | 16 | 6 | 22 |
| St. Abbs and Eyemouth Voluntary Marine Reserve |  | 6 | 6 |
| Strangford Lough Marine Nature Reserve |  | 5 | 5 |
| Sund Rock Marine Preserve | 1 |  | 1 |
| Table Mountain National Park | 4 | 2 | 6 |
| Tawharanui Marine Reserve | 3 |  | 3 |
| Tinderbox Marine Reserve | 5 | 2 | 7 |
| Tsitsikamma National Park | 3 |  | 3 |
| Tulamben | 3 |  | 3 |
| Ushibuka Marine Park | 1 |  | 1 |
| Wadi El Gemal - Hamata Reserve |  | 5 | 5 |
