# Supplementary material for: Reef Fishes at All Trophic Levels Respond Positively to Effective Marine Protected Areas
Source: PLoS One. 2015 Oct 13;10(10):e0140270. doi: 10.1371/journal.pone.0140270 (PMC4603671; doi:10.1371/journal.pone.0140270)
Supplement: S2 Table — PAR-mean, Nitrate, Phosphate, Silicate, Chlomean, SST range and SST mean were obtained from Bio-ORACLE [23]. Pop index was calculated using the quadratic kernel function described by [24]. (DOCX) [file pone.0140270.s005.docx]

**S2 Table.** Covariates used as predictors in linear mixed models. PAR-mean, Nitrate, Phosphate, Silicate, Chlomean, SST range and SST mean were obtained from Bio-ORACLE ([39](#_ENREF_39)). Pop index was calculated using the quadratic kernel function described by ([40](#_ENREF_40)).

| **Code** | **Variable** | **Units** | **Scale** | **Range** |
| --- | --- | --- | --- | --- |
| Pop index | Index of population pressure | index | 4.6 km | 0 - 1 |
| PAR-mean | Photosynthetically-available radiation | Einstein/m^2^/day | 9.2 km | 26.3 - 50.9 |
| SST mean | mean sea surface temperature | ^o^C | 9.2 km | 5.35 - 31.09 |
| SST range | Range of sea surface temperature | ^o^C | 9.2 km | 0.96 - 17.93 |
| Nitrate | Mean nitrate | umol/l | 9.2 km | 0.14 - 15.31 |
| Phosphate | Mean phosphate | umol/l | 9.2 km | 0.02 - 2.26 |
| Silicate | Mean silicate | umol/l | 9.2 km | 0.31 - 37.9 |
| Chlomean | Mean chlorophyll A | mg/m^3^ | 9.2 km | 0.02 - 15.99 |
| Latitude | Site latitude | decimal degrees | 0.0001^o^ | -55.1 - 78.5 |
| Longitude | Site longitude | decimal degrees | 0.0001^o^ | -179 - 175 |
